# An autonomous navigation method for orchard mobile robots based on octree 3D point cloud optimization

**DOI:** 10.3389/fpls.2024.1510683

**Published:** 2025-01-07

**Authors:** Hailong Li, Kai Huang, Yuanhao Sun, Xiaohui Lei, Quanchun Yuan, Jinqi Zhang, Xiaolan Lv

**Affiliations:** ^1^ School of Automation, Nanjing University of Information Science & Technology, Nanjing, Jiangsu, China; ^2^ Institute of Agricultural Facilities and Equipment, Jiangsu Academy of Agricultural Sciences, Nanjing, Jiangsu, China; ^3^ Key Laboratory of Modern Horticultural Equipment, Ministry of Agriculture and Rural Affairs, Nanjing, China; ^4^ Wuxi Yue Tian (YTK) Agricultural Machinery Technology CO., Ltd., Wuxi, China

**Keywords:** octree, autonomous navigation, 3D LiDAR, orchard mobile robot, point cloud optimization

## Abstract

Three-dimensional (3D) LiDAR is crucial for the autonomous navigation of orchard mobile robots, offering comprehensive and accurate environmental perception. However, the increased richness of information provided by 3D LiDAR also leads to a higher computational burden for point cloud data processing, posing challenges to real-time navigation. To address these issues, this paper proposes a 3D point cloud optimization method based on the octree data structure for autonomous navigation of orchard mobile robots. This approach includes two key components: 1) In terms of orchard mapping, the spatial indexing and segmentation features of the octree data structure are introduced. According to the sparsity and density of the point cloud, the 3D orchard map is adaptively divided and the key information of the orchard is retained. 2) In terms of path planning, by using octree nodes as the unit nodes for RRT* random tree expansion, an improved RRT* algorithm based on octree is proposed. Field experiments were conducted in a pear orchard based on this method. The experimental results show that: 1) The overall number of point cloud data points in the map was reduced by approximately 76.32%, while important features, including tree morphology, trellis structure, and road surface information, were fully preserved. 2) When different octree node resolutions were applied, the improved RRT* algorithm demonstrated significant improvements in path generation time, sampling point utilization, path length, and curvature. The lateral tracking error increased as the resolution of octree nodes decreased. At a resolution of 0.20 m, the maximum average lateral tracking error was 0.079 m, indicating strong path trackability. This method exhibits tremendous potential for processing large-scale 3D point cloud data and enhancing path planning efficiency, providing a valuable technical reference for the real-time autonomous navigation of mobile robots in complex orchard environments.

## 1 Introduction

China currently ranks first in the world in both planting area and production of orchards. The fruit industry plays a pivotal role in China’s agricultural economy, ranking just behind grain and vegetables (National Bureau of Statistics of China, 2022). However, the late development of intelligent agricultural machinery in Chinese orchards has resulted in insufficient operational efficiency to address labor shortages and meet the demand for high-quality fruit products (Liu et al., 2022; Zhao et al., 2023). Current application trends show that autonomous navigation technology of orchard mobile robots possesses the capabilities of autonomous perception, positioning, navigation, and decision-making (Jin et al., 2021; Yuan et al., 2023). These robots can autonomously select paths based on the surrounding environment and task requirements, providing technical support for tasks such as unmanned pollination (Gao et al., 2023), weeding (Nørremark et al., 2008; Adrien et al., 2018), spraying (Jones et al., 2019), and fruit harvesting (Xiong et al., 2018). Therefore, introducing intelligent robot autonomous navigation technology is an effective method to improve the production and management efficiency of orchards and address the aforementioned challenges (Dou et al., 2024).

**Figure 1:**
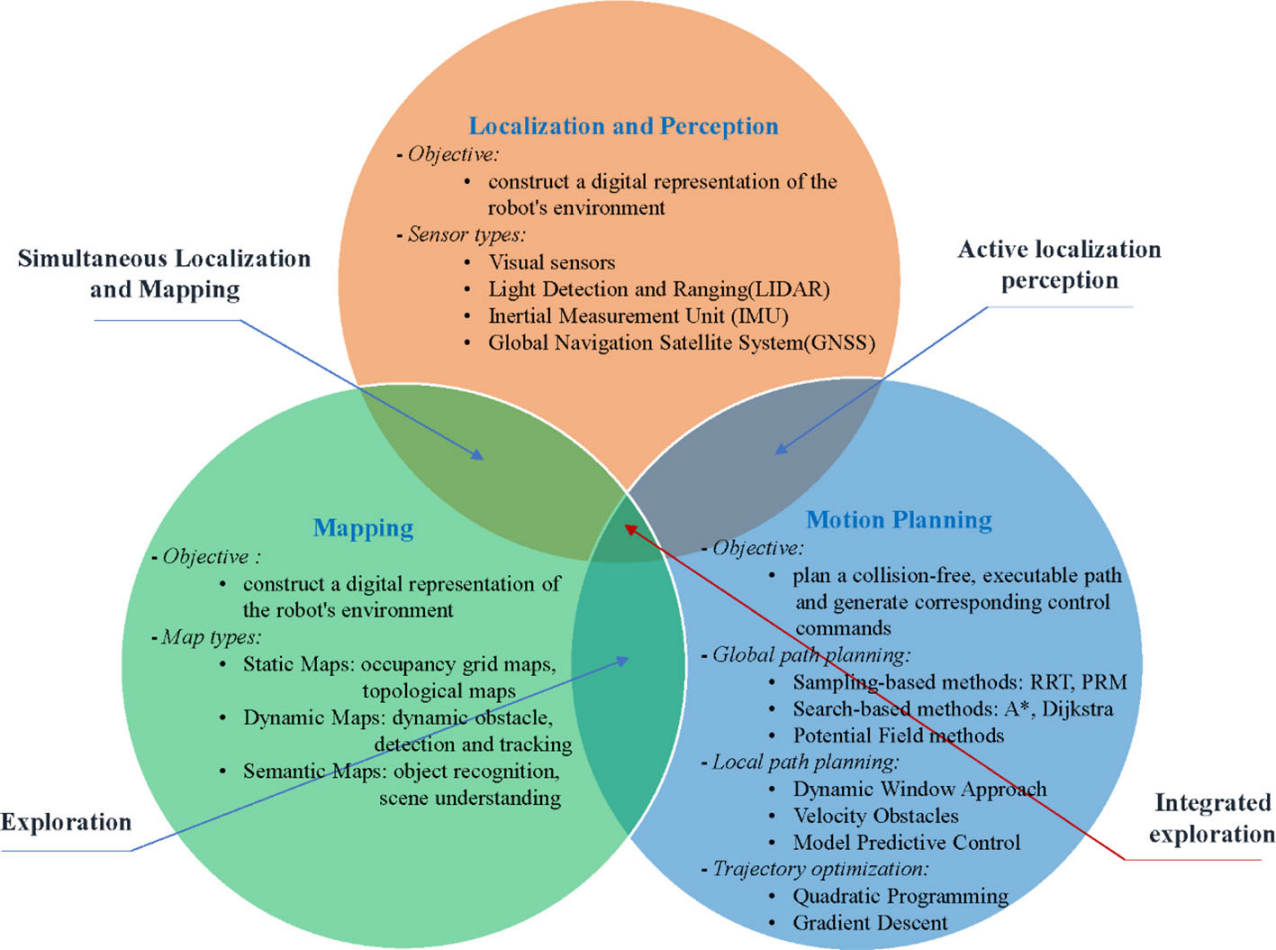
The components of autonomous navigation technology.

Autonomous navigation technology enables robots to perceive their environment, build maps, and plan motion in dynamic environments using onboard sensors with minimal or no human intervention, allowing for autonomous movement (Ravankar et al., 2018; Li and Qiu, 2021). The three related aspects are introduced in detail in **Figure 1**. In orchard environments, widely used navigation methods currently include Global Navigation Satellite System (GNSS), Inertial Navigation System (INS), visual sensors, and LiDAR. **Table 1** provides a comparison of state-of-the-art orchard mobile robot autonomous navigation platforms, highlighting their features, advantages, and limitations.

**Table 1:** Comparison of state-of-the-art focusing on orchard mobile robot autonomous navigation platform.

Author (Year)	Navigation Method	Research Scheme	Existing Problems
Yu et al. (2024)	GNSS	•Developed a tracked orchard sprayer with GNSS positioning, linear path tracking, and motion model integration for automatic navigation.	•GNSS signals are susceptible to obstruction in orchard environments.
Zhang et al. (2022)	GNSS + IMU	•Real-time error correction for IMU utilizing the Kalman error filtering algorithm.	•Lack of semantic information and insufficient support for finegrained tasks.
Han et al. (2021)	Stereo vision camera	•Using U-Net for road segmentation; •Extracting edge information through scanning method; •Fit the navigation path based on B-spline curves.	•It is necessary to readjust the network structure and retrain the model to adapt to new scenarios. Less portability.
Peng et al. (2022)	Depth camera	•Prioritized row-end detection using statistical changes in depth data to ensure robust navigation under GNSS-free conditions.	•Susceptible to visual occlusions caused by dense canopies; accuracy declines with fewer environmental features at row-ends.
Baltazar et al. (2023)	2D LIDAR	•Used 2D LiDAR to continuously detect canopy edges, enabling accurate spray path adjustments.	•2D LiDAR perceives limited information and is not suitable for complex navigation tasks. •It has not been verified in the real-time spraying scene.
Wang et al. (2022)	3D LIDAR	•Constructing an orchard environment map using LiDAR. •Leveraging millimeter-wave radar to aid in obstacle detection with LiDAR.	•The processing of 3D point cloud data involves multiple steps and complex methods.
Liu et al. (2023)	3D LIDAR	•Three steps are employed for preprocessing the 3D point cloud. •Two methods are utilized to extract inter-row navigation lines and subsequently perform complementary fusion on them.	
This paper	3D LIDAR	•Aiming at the large-scale complex 3D point cloud in the orchard, this paper uses octree data structure to simplify the existing processing methods. •An improved RRT* algorithm is proposed by deeply using the processed octree nodes, which improves the sampling efficiency and generates shorter and smoother paths.	

In recent years, navigation systems for orchard environments have seen significant advancements. Yue et al. (2024) developed a GNSS-based automatic navigation driving system specifically for tracked orchard sprayers. They designed a motion model for the sprayer and integrated it with a linear path tracking control method, which uses position and heading deviations as state variables. The tracked platform demonstrated robust automatic navigation capabilities, achieving a maximum straight-line path tracking accuracy of 5.6 cm with a standard deviation of 2.8 cm at an optimal speed of 1.0 m/s. This system effectively meets the requirements for automatic spraying operations in orchards. However, Global Navigation Satellite System (GNSS) signals in orchard environments are easily obstructed by tree canopies, resulting in weakened or even distorted positioning signals. Zhang et al. (2022) used a loosely coupled method to integrate the satellite navigation system with the INS. The system corrected INS errors in real-time using the Kalman error filtering algorithm, based on position and heading angle measurements. Compared with a standalone satellite navigation system, the accuracy of the satellite/Inertial Measurement Unit (IMU) integrated navigation system was significantly improved. However, GNSS+IMU can only provide geometric positions and cannot perceive semantic information the categories of surrounding objects, which is not conducive to the interaction between robots and the environment.

Machine vision technology has been widely researched and applied in the field of autonomous navigation for orchard robots due to its advantages, e.g., low hardware cost and rich visual information (Yasuda et al., 2020; Alkendi et al., 2021). Han et al. (2021) used the U-Net semantic segmentation algorithm to segment, extract, and fit the navigation path between fruit tree rows, effectively eliminating problems, e.g., complex backgrounds and high noise interference in orchard images. Unlike most studies to date that utilize ground structures e.g., tree trunks and canopies in orchards, Peng et al. (2022) developed a navigation system using a depth camera and odometry data to autonomously navigate through vineyard rows without relying on GNSS or artificial landmarks. The system includes a robust row-end detection method, exploiting drastic changes in the statistical distribution of point cloud data sensed by the depth camera. By building a local environment map and utilizing a reactive path tracker, the system enabled safe row-end turning and entry into the next row. This method demonstrated its effectiveness in handling row-end transitions, with a mean row-end detection error of 0.54 m. Although vision-based navigation methods are cost-effective and provide a wealth of navigation information, considering the climate in orchards and the occlusion caused by dense canopies, the impact of frequent light changes on visual sensors cannot be overlooked.

Compared to GNSS and vision-based navigation methods, LiDAR has advantages, e.g., long measurement distance, high accuracy, and rich distance information (Blok et al., 2018; Liu et al., 2021). Baltazar et al. (2023) developed a 2D LiDAR-based navigation system integrated with GNSS for continuous canopy sensing and real-time spray adjustment in orchards. The system detects canopy boundaries, calculates tree volumes, and georeferences canopy data along the sprayer’s trajectory. It demonstrated up to 79% reduction in spray volume compared to traditional methods, ensuring precise and efficient spraying operations. However, the system relies on 2D LiDAR, which is limited in perceiving occluded trunks and dense canopy structures in more complex 3D orchard environments. Addressing these limitations, Wang et al. (2022) utilized 3D LiDAR to perceive environmental information, solving the issue of losing critical spatial data with 2D LiDAR. Their method achieved high-precision detection of tree row information and obstacle perception from multiple directions. Liu et al. (2023) further improved navigation path smoothness by combining dual-source point cloud data from 3D LiDAR at high and low frequencies, enabling complementary fusion of path planning.

Compared to 2D LiDAR, 3D LiDAR has more comprehensive and accurate environmental perception capabilities in autonomous navigation, improving navigation accuracy and safety, further making it more suitable for navigation tasks in complex environments e.g., orchards (He et al., 2018; Zhang et al., 2019). Although the orchard point cloud map obtained by 3D LiDAR scanning can finely depict the 3D structure of the orchard, its data scale is enormous, resulting in requiring not only a large amount of storage space but also high computational power. Moreover, for the application requirements of autonomous navigation, due to the influence of various factors e.g., scanning angle, occlusions, and ground weeds during the actual operation process, the point cloud map contains a large amount of noise points and irrelevant detailed information, causing unnecessary data redundancy and affecting the accuracy and real-time performance of the map (Wang et al., 2018; Yang et al., 2019). In current research on autonomous navigation of orchard robots, filtering methods are used to remove noise points, outliers, and abnormal points, and then sampling methods are used to reduce the data volume. Subsequently, depending on the requirements, clustering, segmentation, feature extraction, and plane fitting methods are chosen to deal with different scenarios (Malavazi et al., 2018; Liu et al., 2021; Xie et al., 2023; Zhang et al., 2024). However, this process is very cumbersome. Taking filtering as an example, it includes different methods e.g., voxel filtering, statistical filtering, nearest neighbor filtering, Gaussian filtering, and adaptive filtering. Selecting the appropriate method and ensuring the processing effectiveness and efficiency is a significant challenge.

To address the aforementioned research problem of processing large-scale 3D point cloud data in orchards, the octree data structure has unique advantages in managing and processing large-scale point cloud data due to its efficient data compression, precise spatial partitioning, and fast data indexing capabilities. However, it has not been well applied in the field of autonomous navigation for mobile robots in orchards. Therefore, this paper proposes a navigation scheme based on 3D LiDAR, while utilizing octree to optimize the collected 3D point cloud data of the orchard and construct an orchard map. Then, based on the generated octree nodes, an improved RRT* algorithm is proposed to accelerate the generation of navigation paths. The pure pursuit algorithm is used to track the navigation path. Finally, field experiments are conducted to verify the performance of the navigation system. The innovation proposed in this study primarily manifests in the following aspects:

1. This study introduces the efficient spatial partitioning and segmentation capabilities of the octree data structure for processing 3D point cloud data in orchards, thereby optimizing the workflow for handling such complex data within the orchard domain.
2. A novel RRT* algorithm enhanced by both octree and an adaptive step size adjustment strategy is proposed in this research. By utilizing octree nodes as the foundational units for tree expansion and carefully examining the relationship between step size thresholds and node sizes, a significant improvement in both path generation speed and quality is achieved.

The subsequent sections of this paper are organized as follows: Section 2 elaborates on the materials and methods employed in this study, encompassing hardware tools, software systems, control principles, and a comprehensive exposition on the characteristics of the octree data structure and the core framework of the enhanced RRT* algorithm based on octree. Section 3 delineates the experimental scenarios and methodologies utilized in this study. Section 4 focuses on an in-depth analysis of the optimization impacts on 3D point clouds leveraging octree, along with the evaluation of path generation speed and quality across varying node resolutions. Sections 5 and 6 encapsulate the significance and constraints of this research, while outlining prospective directions for future research pursuits.

## 2 Materials and methods

### 2.1 Autonomous navigation platform for orchard mobile robots

**Figure 2:**
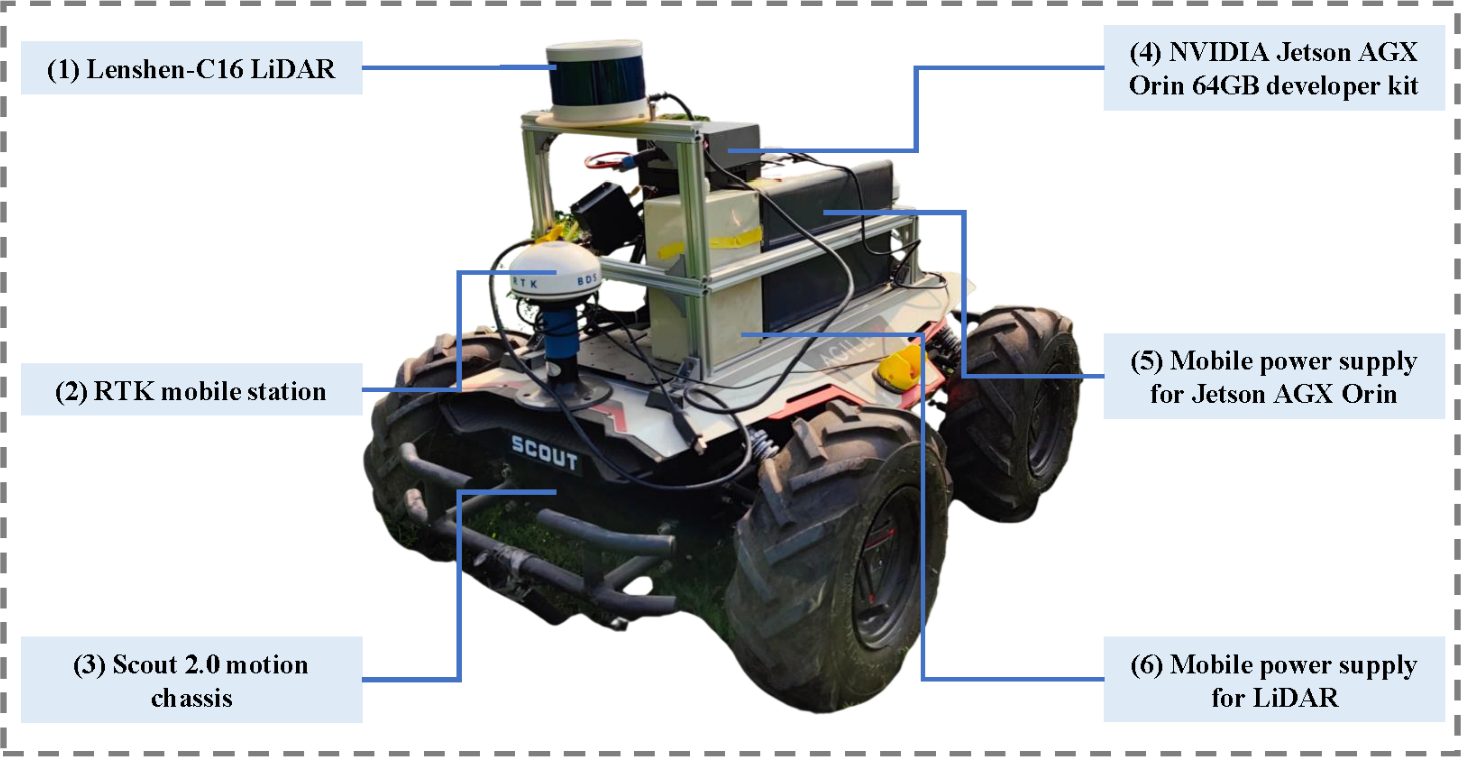
Orchard mobile robot autonomous navigation platform.

The autonomous navigation platform for mobile robots employed in this study is illustrated in **Figure 2**. The platform primarily comprises six components: (1) a 3D LiDAR, (2) an onboard Real-Time Kinematic Global Navigation Satellite System (RTK-GNSS), (3) a mobile chassis, (4) an onboard central computer, (5) a mobile power supply for Jetson AGX Orin, and (6) a mobile power supply. **Table 2** presents a comprehensive overview of the working parameters for the key components.

**Table 2:** Key Components and parameters of orchard mobile robot autonomous navigation platform.

No	Name	Main Work Parameters	Company	Manufacturing Location
(1)	LiDAR	• Lidar Channels: 16 • Measurement Range (m): 0~200 • Velocity Accuracy (cm): ± 3 • Scanning Frequency (Hz): 5,10,20 (optional) • Vertical Angular Resolution (°): 2 • Horizontal Angular Resolution (°): 0.09,0.18,0.36	Leishen LIDAR	Shenzhen, China
(2)	RTK-GNSS	• Horizontal positioning accuracy in fixed solution: ± 1cm • Initialization time <10 s • Data output frequency: 30Hz	QFRTK	Shenzhen, China
(3)	Motion chassis	• Dimensions (Length× Width× Height): 930mm×699mm×349 mm • Wheelbase: 498mm • Maximum Moving Speed: 1.5 m/s • Maximum Off-road Angle: 30°	AGILE.X	Shenzhen, China
(4)	Jetson AGX Orin	• AI Performance: 275 TOPS • GPU: 2048-core NVIDIA Ampere architecture GPU with 64 Tensor Cores • CPU: 12-core Arm^®^ Cortex^®^-A78AE v8.2 64-bit CPU 3MB L2 + 6MB L3 • Memory: 64GB 256-bit LPDDR5 204.8GB/s	NVIDIA	Santa Clara, California, USA

**Figure 3:**
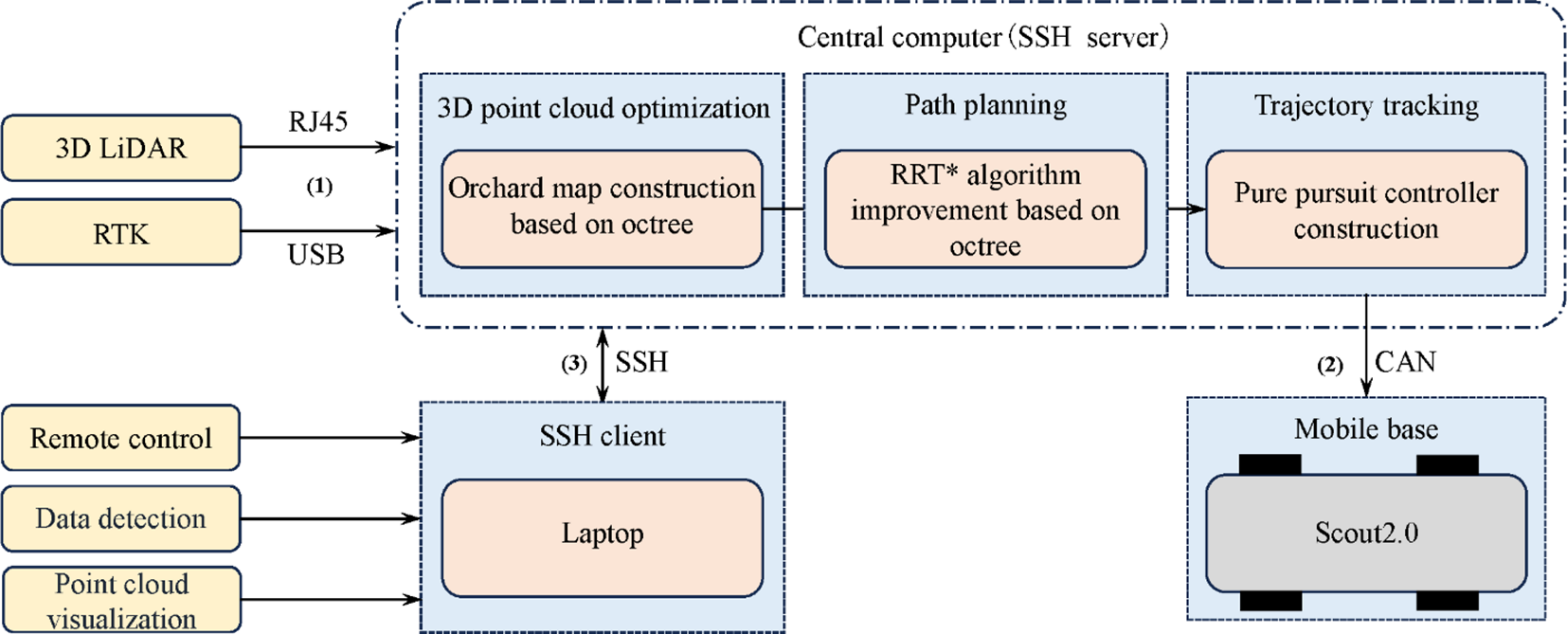
Control flow chart of mobile robot.

In the software system of the above platform, the Ubuntu 20.04 LTS operating system is utilized on Jetson AGX Orin. The Robot Operating System (ROS) Noetic version, Ceres non-linear optimization library, and Point Cloud Library (PCL) are utilized for further development. The complete control framework of the above platform is depicted in **Figure 3**:

1. The orchard environment is scanned by the LiDAR at a frequency of 10 Hz to acquire point cloud information, further being transmitted to the central computer (Jetson AGX Orin embedded computing platform) via Ethernet. Point cloud optimization, path planning and path tracking are performed on the central computer, in which the control commands are generated.
2. The control commands are sent to the mobile chassis through CAN communication. The received control commands are converted by mobile classis into the required speed commands for motor operation, ultimately enabling the mobile robot to achieve autonomous movement.
3. The laptop and the central computer are integrated through SSH communication to establish a distributed control framework, facilitating data monitoring and control.

### 2.2 Octree-based optimization method for 3D point cloud in orchards

**Figure 4:**
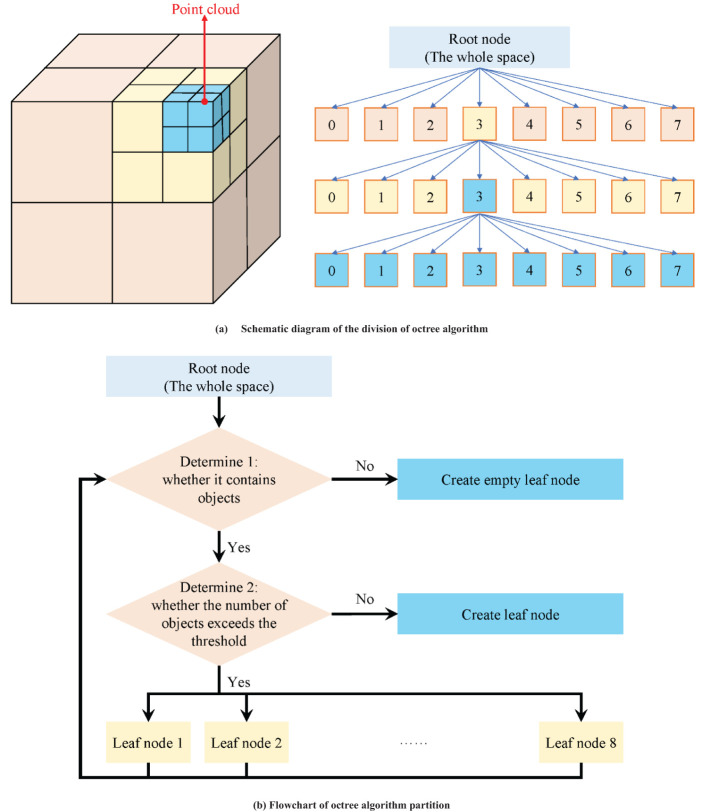
Schematic diagram and partition process of octree algorithm. **(A)** Schematic diagram of the division of octree algorithm. **(B)** Flowchart of octree algorithm partition.

As a multi-level data structure based on spatial partitioning, octree efficiently organizes orchard 3D point cloud data by recursively dividing the 3D space into eight equal sub-cubes, also referred to as leaf nodes (Huang et al., 2020; Que et al., 2021; Fu et al., 2022; Wang, 2023). Specifically, the octree algorithm considers the entire 3D point cloud space as the root node and constructs eight child nodes from top to bottom by setting appropriate recursion depth and threshold values. If a node still contains point cloud data, it is further subdivided into eight child nodes until the maximum recursion depth or minimum node size is attained. **Figure 4** illustrates the detailed partitioning process and provides a schematic representation of the octree algorithm.

In practical applications, due to the dynamic changes in the orchard environment and the presence of noise, sometimes a node may contain point cloud data and sometimes not. Therefore, nodes within the entire point cloud space can have three possible states: free, unknown, and occupied. To explicitly express the “unknown” state, a floating-point number between (0, 1) is typically used to represent the probability of a node being occupied. A value of 0.5 indicates that the node’s state is “uncertain”, with higher values suggesting a greater likelihood of the node containing point cloud data, and lower values indicating a lower likelihood. According to the derivation of octree theory (Wurm et al., 2010; Hornung et al., 2013), assuming that the observed data at times *t=1, 2,…, T* are *d_1_, d_2_,…, d_T_*, the information recorded by the *n^th^* leaf node is:


(1) $$P\left(n|d_{1:T}\right)={\left[1+\frac{1-P\left(n|d_{T}\right)}{P\left(n|d_{T}\right)}\frac{1-P\left(n|d_{1:T-1}\right)}{P\left(n|d_{1:T-1}\right)}\frac{P\left(n\right)}{1-P\left(n\right)}\right]}^{-1}$$

In the Equation 1:

-
$P\left(n|d_{1:T}\right)$ : The posterior probability of the *n^th^* leaf node being occupied given the observed data *d_1_* to *d_T_*.
-
$P\left(n|d_{T}\right)$ : The posterior probability of the *n^th^* leaf node being occupied given the observed data *d_T_* at the current time *T*.
-
$P\left(n|d_{1:T-1}\right)$ : The posterior probability of the *n^th^* leaf node being occupied given the observed data *d_1_* to *d_T-1_*.
-
P(n) : The prior probability of the *n^th^* leaf node being occupied, i.e., the initial estimate of the *n^th^* node being occupied without any observed data.

This formula is essentially an application of Bayes’ theorem. It tells us that given the observed data *d_T_* at the current time and the posterior probability $P\left(n|d_{1:T-1}\right)$ from the previous time, we can calculate the updated posterior probability $P\left(n|d_{1:T}\right)$ of node *n* at time *T*. By recursively applying this formula at each time step, we can continuously fuse new observed data and update the occupancy probability of each leaf node in the octree, thereby achieving real-time modeling of the orchard environment.

In order to map the probabilities between 0 and 1 to the range of real numbers from negative infinity to positive infinity, allowing probability values to be linearly combined and compared, the logit transformation is used. The logit transformation is a mathematical transformation that converts probabilities into log-odds, commonly used in modeling and analysis of binary classification problems. Equation 2 is the expression for the logit transformation:


(2) $$\alpha =logit\left(P\right)=log\left(\frac{P}{1-P}\right)$$

α is the log-odds, which refers to the natural logarithm of the ratio of the probability of an event occurring to the probability of it not occurring. If *L* represents the log-odds of each leaf node, the probability update formula after the logit transformation becomes:


(3) $$L\left(n|d_{1:T}\right)=L\left(n|d_{1:T-1}\right)+L\left(n|d_{T}\right)$$

(4) $$P=logit^{-1}\left(\alpha \right)=\frac{1}{1+e^{-\alpha }}$$

From Equations 3 and 4, it is evident that whenever the octree information needs to be updated, it is only necessary to add new information and then convert it back to the original probability. Through this “divide and conquer” strategy, the octree can adaptively refine the dense point cloud regions in the orchard while maintaining a lower resolution for sparse point cloud regions. This approach greatly reduces the amount of point cloud data while retaining important environmental features such as tree morphology and trellis structures in the orchard.

To utilize the spatial partitioning characteristics of the octree for removing ground weeds, an appropriate height threshold must be set based on the characteristics of the orchard environment. This threshold should be slightly higher than the average height of the road surface but not so high that it misclassifies the point cloud data of trees and trellis structures as weeds. During the construction of the octree, the height values of the point cloud data contained within each octree node are checked. If the heights of all points within the node fall within the aforementioned range, the node is considered to satisfy the road surface characteristics, and the node and its child nodes are retained; otherwise, it is classified as ground weeds and removed. By employing this method, the octree can effectively retain road surface information while removing ground weeds, providing more accurate and reliable environmental information for autonomous navigation and operation tasks.

### 2.3 Path planning method of improved RRT* algorithm based on octree

The Rapidly-exploring Random Tree (RRT) family of algorithms employs random sampling to expand search trees, effectively addressing path planning challenges in high-dimensional spaces. They exhibit characteristics such as fast path generation, easy extensibility, and probabilistic completeness, making them widely used in robot motion planning and autonomous driving (Pandian and Noel, 2020; Zhang et al., 2020; Yu et al., 2024). The RRT* algorithm introduces the process of re-selecting parent nodes and rewiring based on the traditional RRT algorithm, optimizing path quality and asymptotic optimality to a certain extent, albeit at the cost of increased computational complexity.

**Figure 5:**
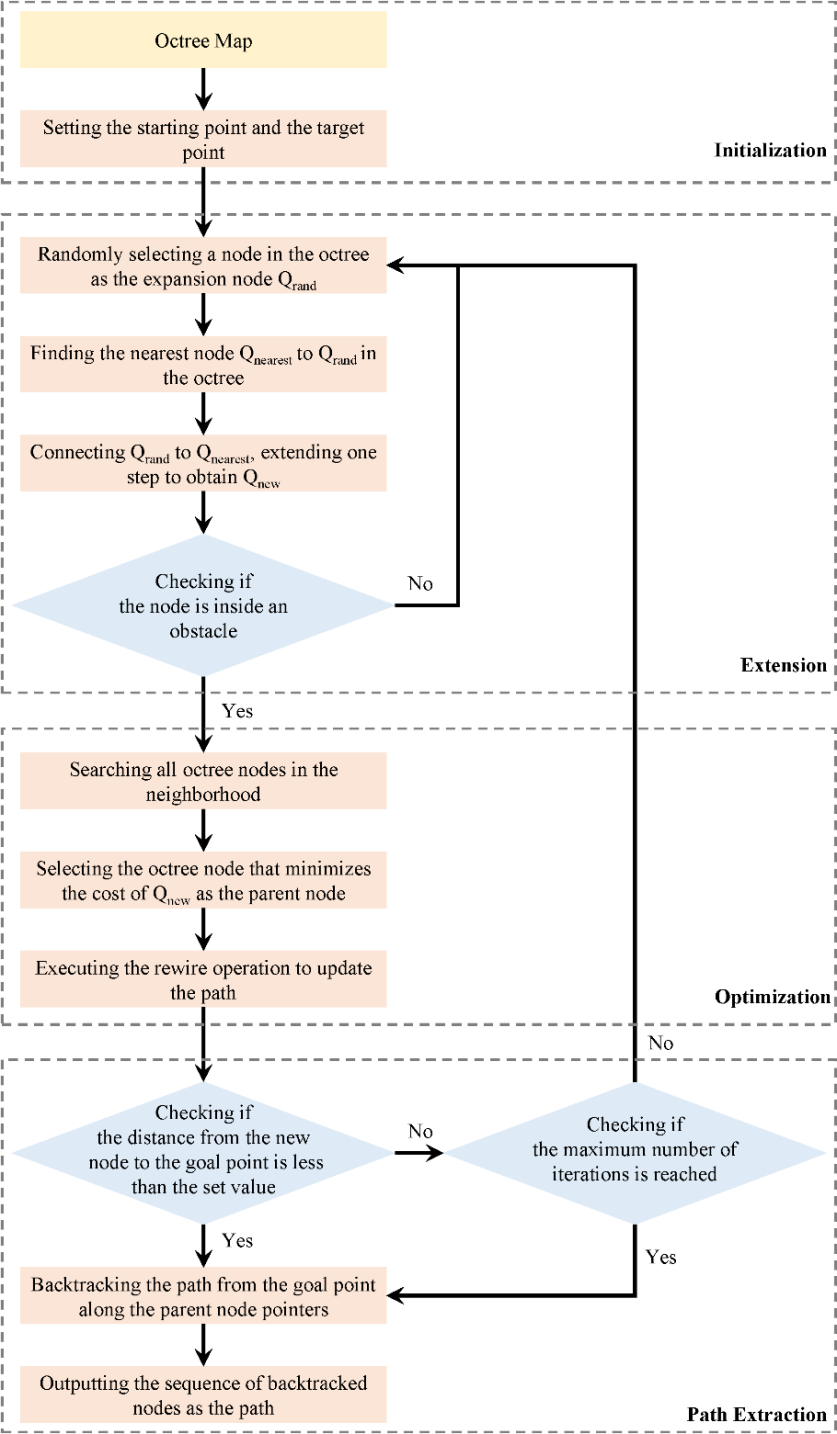
Flowchart of the improved RRT* algorithm based on octree.

While orchard inter-row spaces exhibit a certain degree of regularity, deterministic path-planning methods may lack flexibility in addressing real-world uncertainties, such as obstacles, irregular tree arrangements, and dynamic terrain changes. Therefore, this paper proposes an improved RRT* algorithm that leverages the octree-processed point cloud map for global path planning. The use of random sampling allows this method to flexibly handle the complex environmental features of orchards, while the octree’s spatial partitioning significantly enhances sampling efficiency by reducing unnecessary sample points. This method integrates an adaptive step-size adjustment strategy to balance sampling precision and path-planning speed, making it particularly suitable for large-scale 3D orchard environments (as shown in **Figure 5**). The algorithm mainly consists of the following four steps:

(1) Initialization: Defining the three-dimensional space, starting point, and target point. Choosing an appropriate node resolution based on the size of the environment and the distribution of obstacles. Creating an octree and inserting the starting point as the root node.
(2) Expansion: Randomly selecting a node from the octree as the expansion node. Within the octree node where the expansion node is located, randomly sampling a new position. Utilize the efficient query feature of the octree to check whether the new position is inside an obstacle. If it is, discard the position and resample. Find the node nearest to the new position in the octree as the parent node. Create a new node and connect it to the parent node while inserting it into the octree.
(3) Optimization: Performing a neighborhood search by leveraging the spatial partitioning property of the octree to search only the nodes adjacent to the octree node where the new node is located, instead of searching the entire tree. Reducing the search scope. Attempting to connect the new node to each neighboring node. Updating the parent node of the new node if the path cost is lower after the connection.
(4) Path Extraction: Checking if the distance between the new node and the target point is smaller than a set threshold; if so, considering a feasible path to be found, and terminating the algorithm. Terminating the algorithm if the maximum number of iterations or other termination conditions are reached. Backtracking from the target point along the parent node pointers to the starting point. Using the sequence of backtracked nodes as the final path.

**Figure 6:**
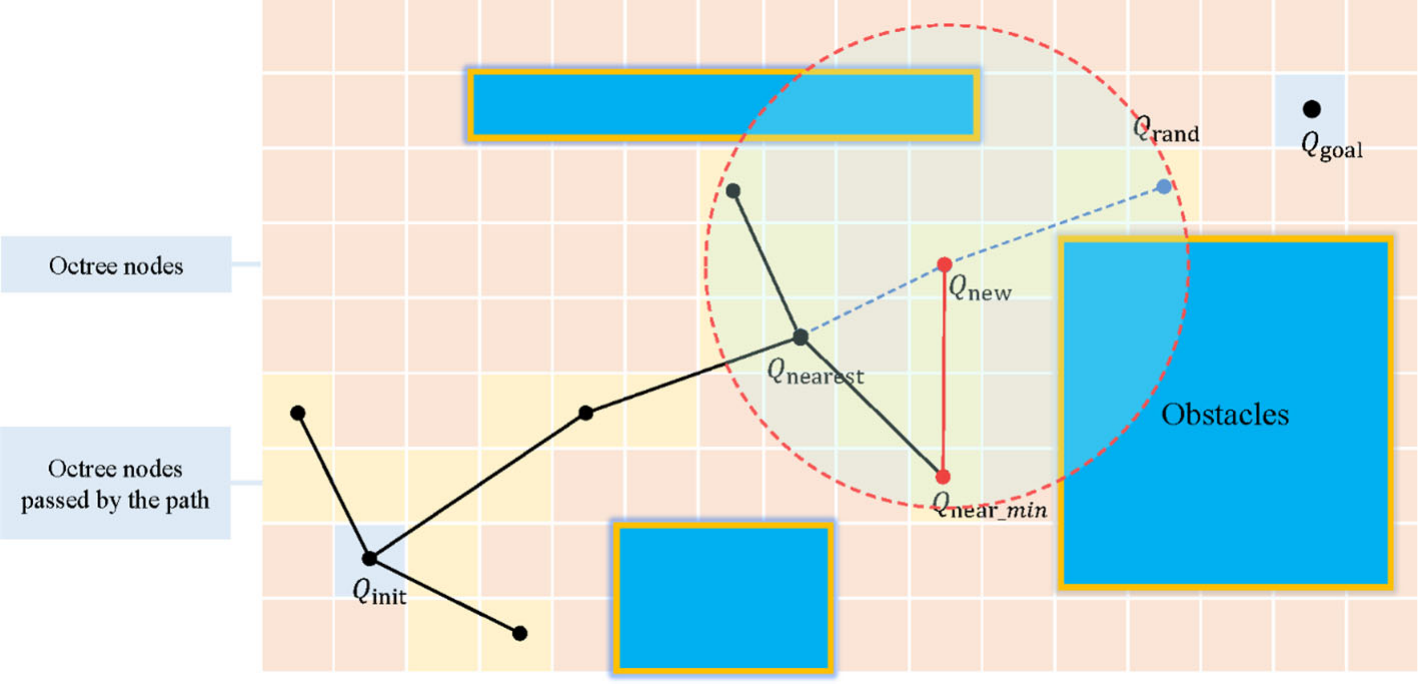
Schematic diagram of the expansion process of the improved RRT* algorithm based on octree. Q_init_, starting point; Q_goal_, target point; Q_rand_, random sampling point; Q_new_, new node; Q_nearest_, original parent node; Q_near_min_, re-selected parent node. In the figure, the red dashed circle represents the defined radius range for finding all nearby nodes Q_near_. The black solid line represents the original path, and the red solid line represents the path after rewire.

**Figure 7:**
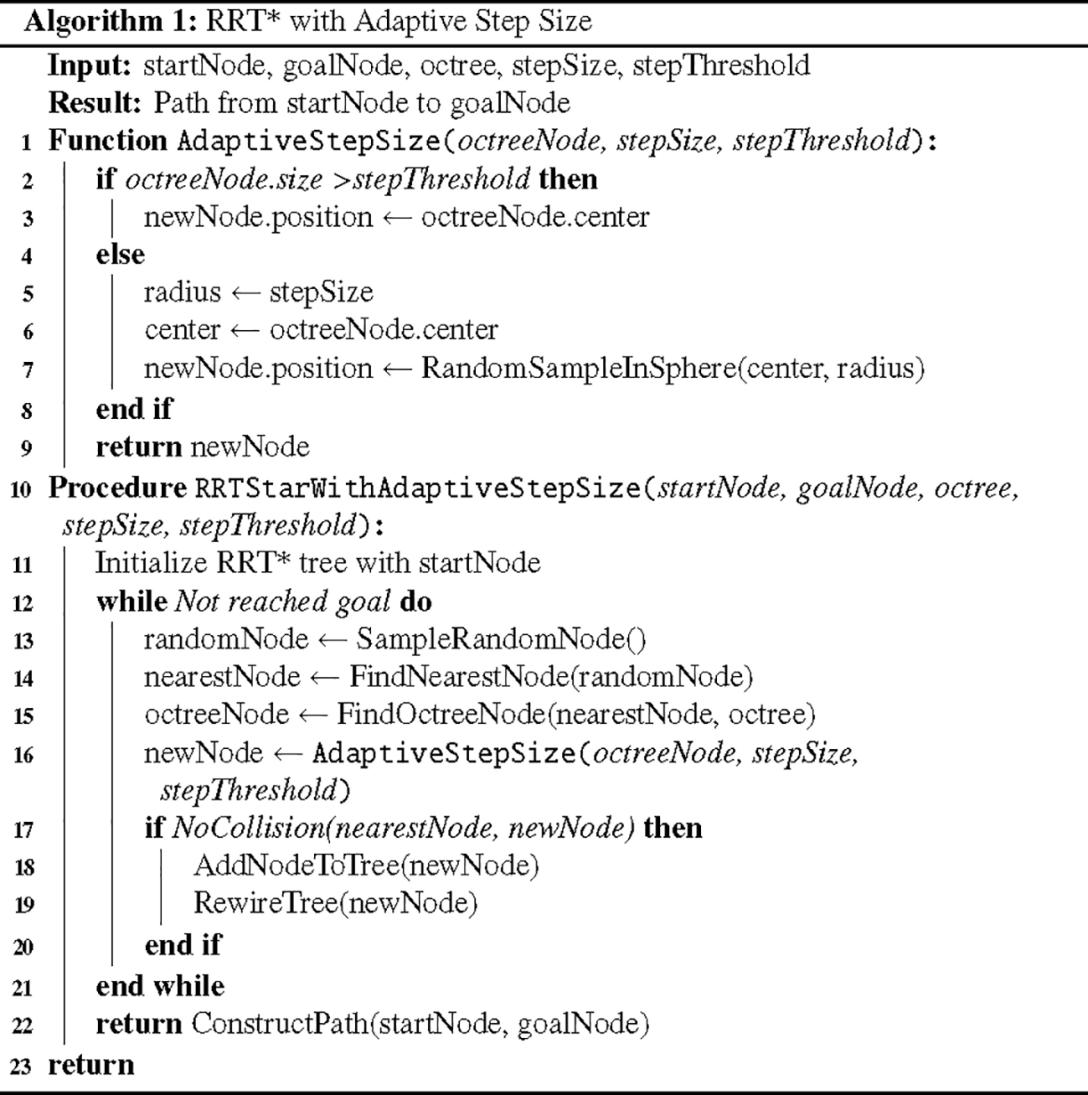
Pseudocode for adaptive step-size adjustment algorithm.

When using octree nodes as the unit nodes of the search tree in the RRT* algorithm, the expansion process is shown in **Figure 6**. To balance the octree resolution and the step size of the RRT* algorithm, this method introduces an adaptive step size adjustment strategy. The pseudocode is shown as **Figure 7**. When the size of an octree node is larger than a preset step size threshold, the node center is directly used as the new node position; conversely, when the node size is less than or equal to the threshold, a new node position is randomly sampled within a range centered at the node center with a radius equal to the step size. This allows for appropriately increasing the step size in regions with lower octree resolution to accelerate tree expansion, while maintaining the original step size in regions with higher resolution to ensure path refinement.

By combining the above-mentioned octree sampling strategy with adaptive step size adjustment, this method inherits the probabilistic completeness and asymptotic optimality of the RRT* algorithm while fully utilizing the hierarchical structure and neighborhood information of the octree. This allows for more effective coverage and exploration of the entire space, reducing redundant and unnecessary sampling, and simultaneously generating shorter and smoother paths.

### 2.4 Path tracking

**Figure 8:**
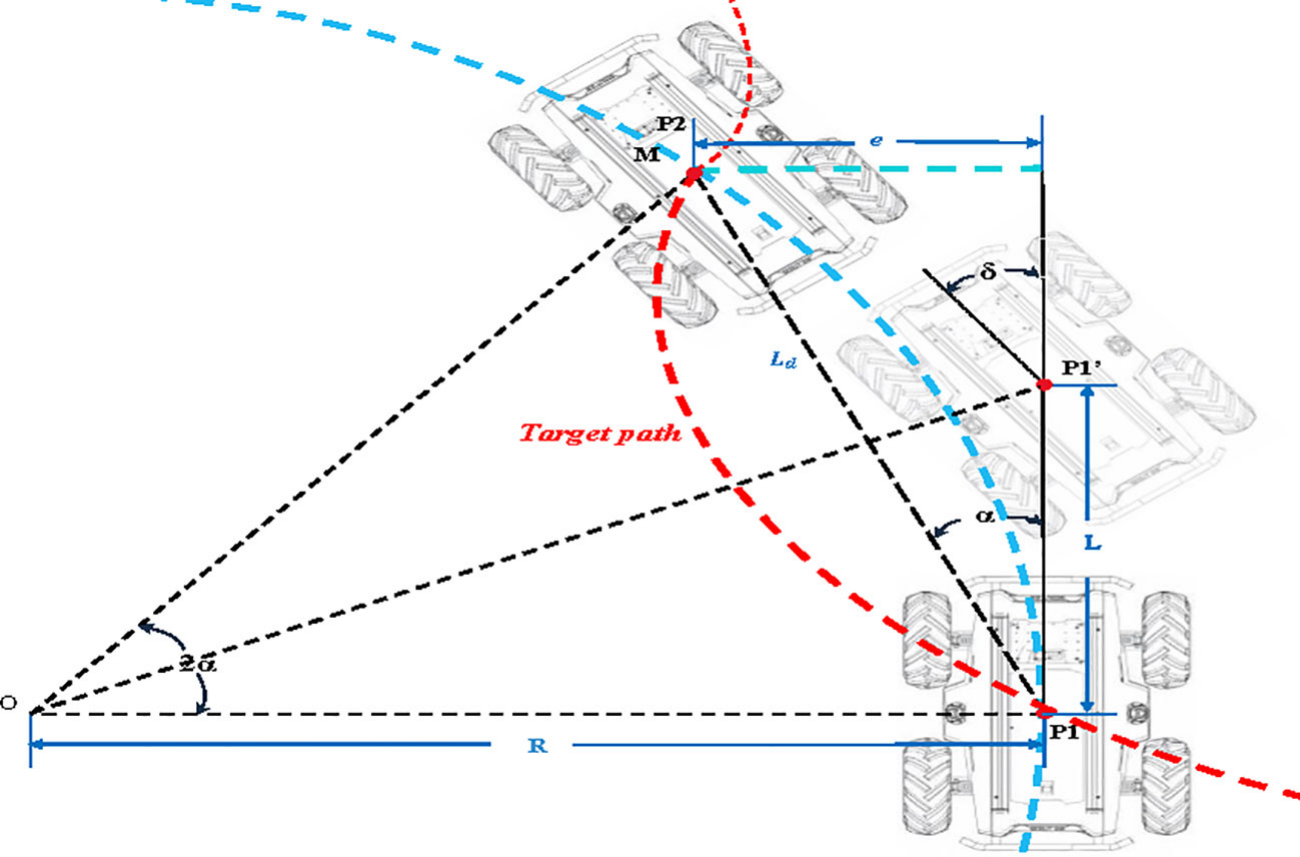
Schematic diagram of pure tracking algorithm. In the figure, *P_1_* denotes the initial pose of the robot; *P_1_’* indicates the pose during turning; *P_2_* represents the target pose of the robot; Point *M* signifies the lookahead position; *L_d_* denotes the lookahead distance; the red dashed line indicates the target path; the blue dashed line represents the approximate path; *R* stands for the current turning radius; *L* is the distance traveled by the robot to reach the turning point; *e* denotes the lateral deviation at the current moment; *α* represents the required heading angle at the current moment; *δ* signifies the heading angle of the robot when reaching the turning point.


Mobile robots in orchards usually travel at low speeds, and the path curvature generally does not change significantly. The mobile robot’s drive-by-wire chassis used in this study satisfies the differential drive kinematics model, as shown in **Figure 8**. Therefore, we consider using a simple and efficient pure pursuit algorithm for path tracking of the optimized path. In the **Figure 8**, the red dashed line represents the target path. At the look-ahead distance *L_d_* on the target path, the current tracking point *M* is selected. The current robot’s driving path can be approximately considered as the circular arc corresponding to the chord *L_d_*, where the radius *R* is the current turning radius. Based on trigonometric functions, the relationship between the look-ahead distance and the turning radius *R* is derived as follows:

(5) $$\frac{L_{d}}{\sin2\alpha }=\frac{R}{\cos\alpha }$$

(6) $$R=\frac{L_{d}}{2\sin\alpha }$$

The above Equations 5 and 6 indicates that for mobile robots, the current linear velocity at a given moment is known, and it is only necessary to solve for the heading angle δ required for the robot to turn. According to the trigonometric function relation, the following formula can be obtained:


(7) $$\tan\delta =\frac{L}{R}$$

Substituting Equation 6 into Equation 7 and introducing the time dimension, the expression of the control quantity *δ* is obtained as follows:


(8) $$\delta \left(t\right)=\tan^{-1}\left(\frac{2L\sin\left(\alpha \left(t\right)\right)}{L_{d}}\right)$$

The ultimate controlled variable of the pure pursuit algorithm is the steering angle *δ*. However, for practical applications, it is essential to understand the parameters that require adjustment. From Equation 8, it is evident that the primary factor influencing the control variable *δ* is the selection of the look-ahead distance *L_d_*. To better comprehend the impact of the look-ahead distance on the pure pursuit algorithm controller, the curvature of the corresponding circle can be analyzed. Based on trigonometric relationships, the following equation can be derived:


(9) $$\sin\alpha =\frac{e}{L_{d}}$$

In the Equation 9, *e* represents the lateral deviation at the current moment. Substituting Equation 9 into Equation 6 yields the curvature of the curvature circle:


(10) $$K=\frac{1}{R}=\frac{2}{{\left(L_{d}\right)}^{2}}e$$

As such, it can be inferred that the larger the *L_d_*, the smaller the curvature, resulting in smoother robot adjustments but potentially leading to delayed responses. Conversely, a smaller *L_d_* yields more precise tracking but may also introduce oscillations.

## 3 Experiment design

**Figure 9:**
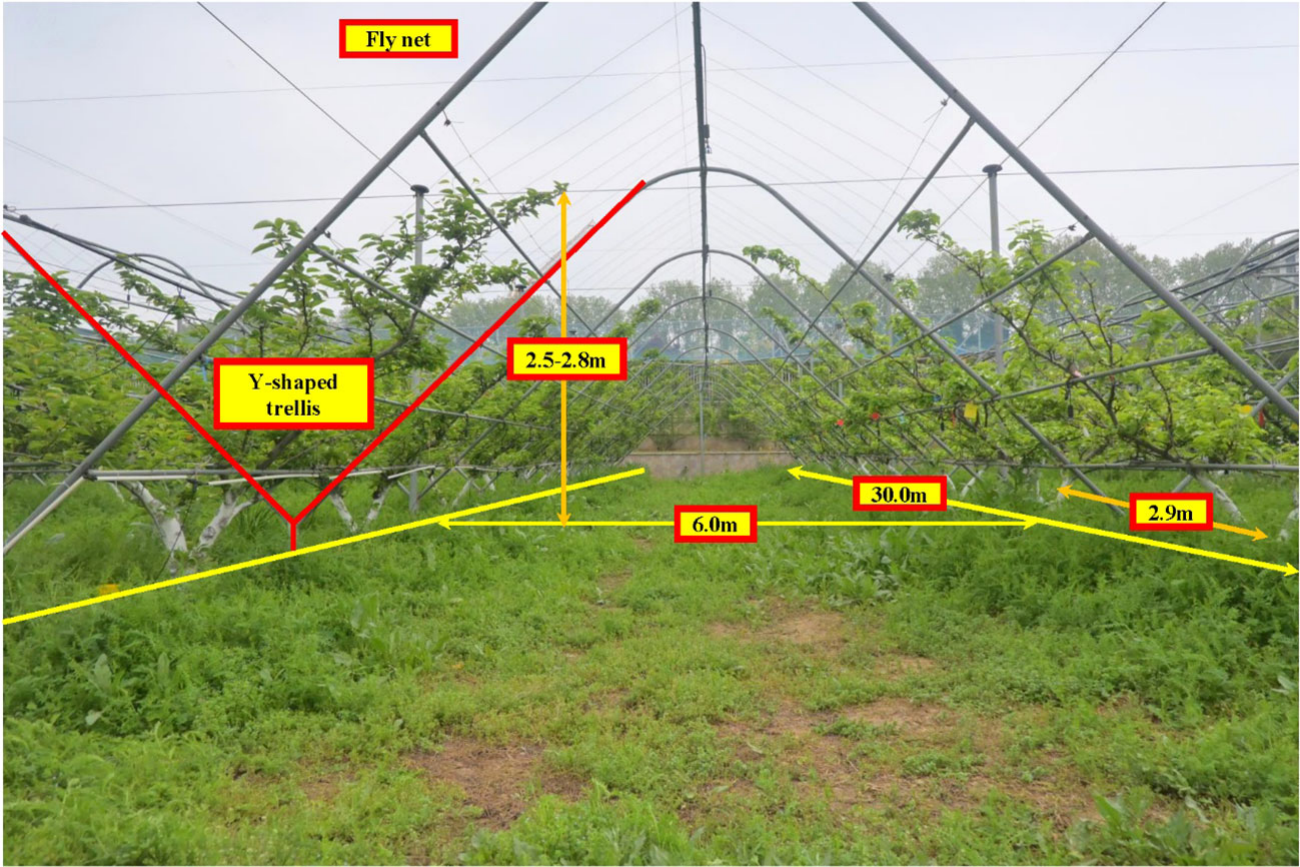
“Y”-shaped trellis orchard.

The experiments in this study were conducted in a pear orchard at the Jiangsu Academy of Agricultural Sciences, located in the Xuanwu district, Nanjing, Jiangsu province, China (N: 118°52’15.42”, E: 118°52’15.42”). As shown in **Figure 9**, the experiments took place on April 14th, 2024. The orchard employs a standard Y-shaped trellis system, with tree rows measuring 30.0 m in length, a row spacing of 6.0 m, and a plant spacing of approximately 2.9 m. Tree heights range between 2.5 m and 2.8 m, and the widths are consistent across the orchard. The trees are relatively uniform in size, and the rows are arranged in straight lines. The road surface between the rows is generally level, meeting the experimental requirements of this study.

To facilitate the operation and real-time monitoring of the robot’s performance, an SSH-based communication framework was established between a laptop and the embedded computing platform, enabling distributed remote control. During the experiment, the scanning distance of the LiDAR was limited to adequately cover the fruit trees on both sides of the current row. This adjustment aimed to reduce noise interference and improve processing speed. The specific experimental steps are as follows:

1. After establishing equipment connections, the LiDAR-scanned point cloud data from each frame was transferred to the octree for optimization. The processed point cloud data was then visualized in RVIZ. In the experimental scenario, the height of the weeds surrounding the fruit trees typically ranged between 0.18 and 0.25 m. To preserve the structural integrity of the fruit trees, a height threshold of 0.20 m was applied during the experiment to filter out weeds.
2. To evaluate the practical performance of the improved RRT* algorithm based on octree, three different octree node resolutions were selected. During the experiment, the robot was positioned at the starting point of the centerline of a tree row. Both the improved RRT* algorithm and the traditional RRT* algorithm were tested for path planning within a single row. Each algorithm was run 30 times, and the average results were calculated. As the RRT* algorithm added new nodes during each iteration, the coordinates of the newly added nodes were published using the “Publisher” feature of ROS with built-in message types. These published path point messages were subscribed to and stored by a new ROS node via the “Subscriber” for subsequent analysis.
3. The experimental orchard had an inter-row spacing of approximately 6.0 m, with minimal RTK signal interference from canopy occlusion. During the experiment, the real-time pose information provided by the RTK positioning module was used to track the mobile robot’s actual position. The robot’s lateral tracking error at different positions was then calculated based on this data.

## 4 Results

### 4.1 Optimization experiment of 3D point cloud map

**Figure 10:**
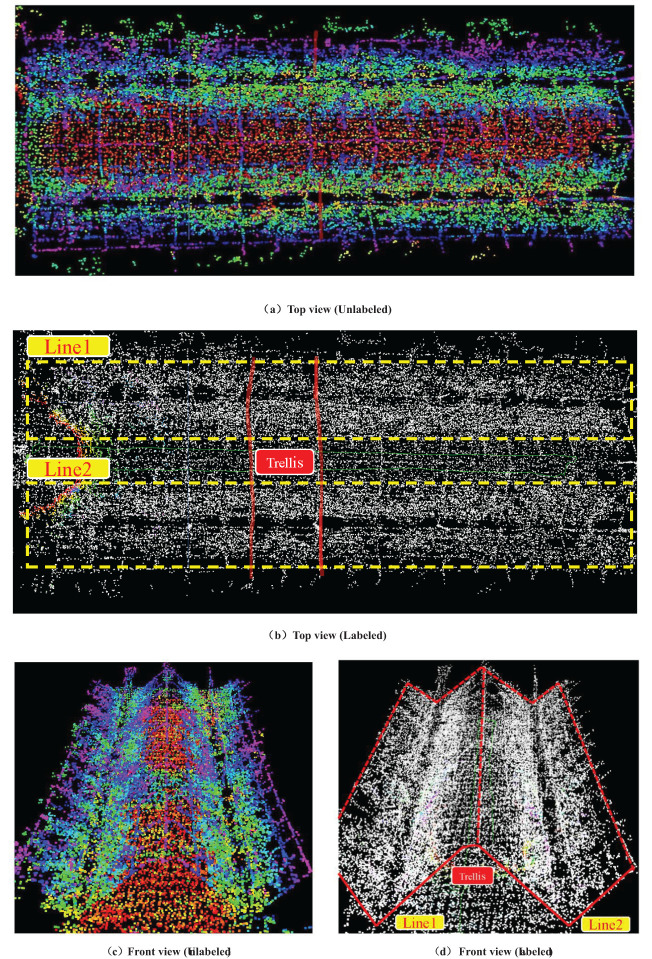
Orchard map optimized by octree 3D point cloud. **(A)** Top view (Unlabeled). **(B)** Top view (Labeled). **(C)** Front view (Unlabeled). **(D)** Front view (Labeled).

**Figure 11:**
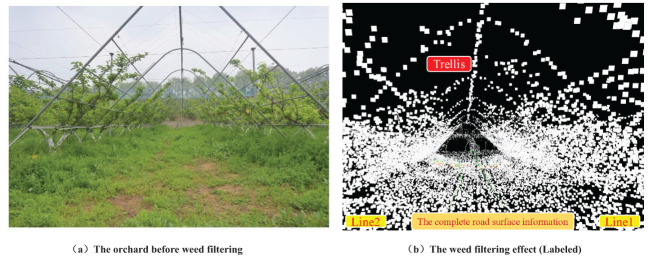
The weed filtering effect based on the octree data structure. **(A)** The orchard before weed filtering. **(B)** The weed filtering effect (Labeled).

The orchard map optimized by octree is illustrated in **Figure 10**, which also illustrates the point cloud map after height rendering is disabled for better annotation and observation. The original point cloud contains approximately 265,000 points per frame. After optimization using octree, each frame comprises about 62,752 points. Compared to the original point cloud map, the number of points in the octree-optimized point cloud has decreased by approximately 76.32%. As shown in **Figures 10A–C**, the fruit tree shape and the “Y” shaped trellis structure can be clearly observed, indicating that this method can effectively retain the important environmental features of the orchard while reducing the number of point clouds, which is beneficial for navigation and other operational tasks. The weed filtering results are presented in **Figure 11**. It is evident that the weed removal effect is significant, and while filtering the weed nodes, it also preserves relatively complete road surface information, providing sufficient road surface nodes for subsequent path planning and expansion.

### 4.2 Path planning and path tracking experiment

**Table 3** presents the average performance metrics of the improved RRT* algorithm based on octree compared to the traditional RRT* algorithm. The results indicate that the octree-based improved RRT* algorithm outperforms the traditional algorithm across all evaluated aspects. Specifically, the average path generation time increased by over 44.98%, the average sampling point utilization rate improved by more than 19.02 percentage points, the average path length decreased by over 9.20%, and the average curvature reduced by more than 51.16%. Additionally, as the octree node resolution decreases, the path response speed improves, and the sampling point utilization rate increases. Conversely, higher octree node resolutions result in more accurate path planning but come at the expense of increased computation time and a slight decrease in sampling point utilization efficiency.

**Table 3:** Comparison of algorithm performance.

Algorithm		Average Path Generation Time (s)	Average Utilization Rate of Sampling Points (%)	Average Path Length (m)	Average curvature of path (m^-1^)
RRT*		14.05	11.37	37.03	0.86
Informed-RRT*		7.02	20.52	33.86	0.65
Improved RRT* based on octree	Resolution:0.20m	4.26	45.21	33.62	0.42
	Resolution:0.10m	5.91	37.22	32.48	0.29
	Resolution:0.05m	7.73	30.39	30.94	0.17

**Figure 12:**
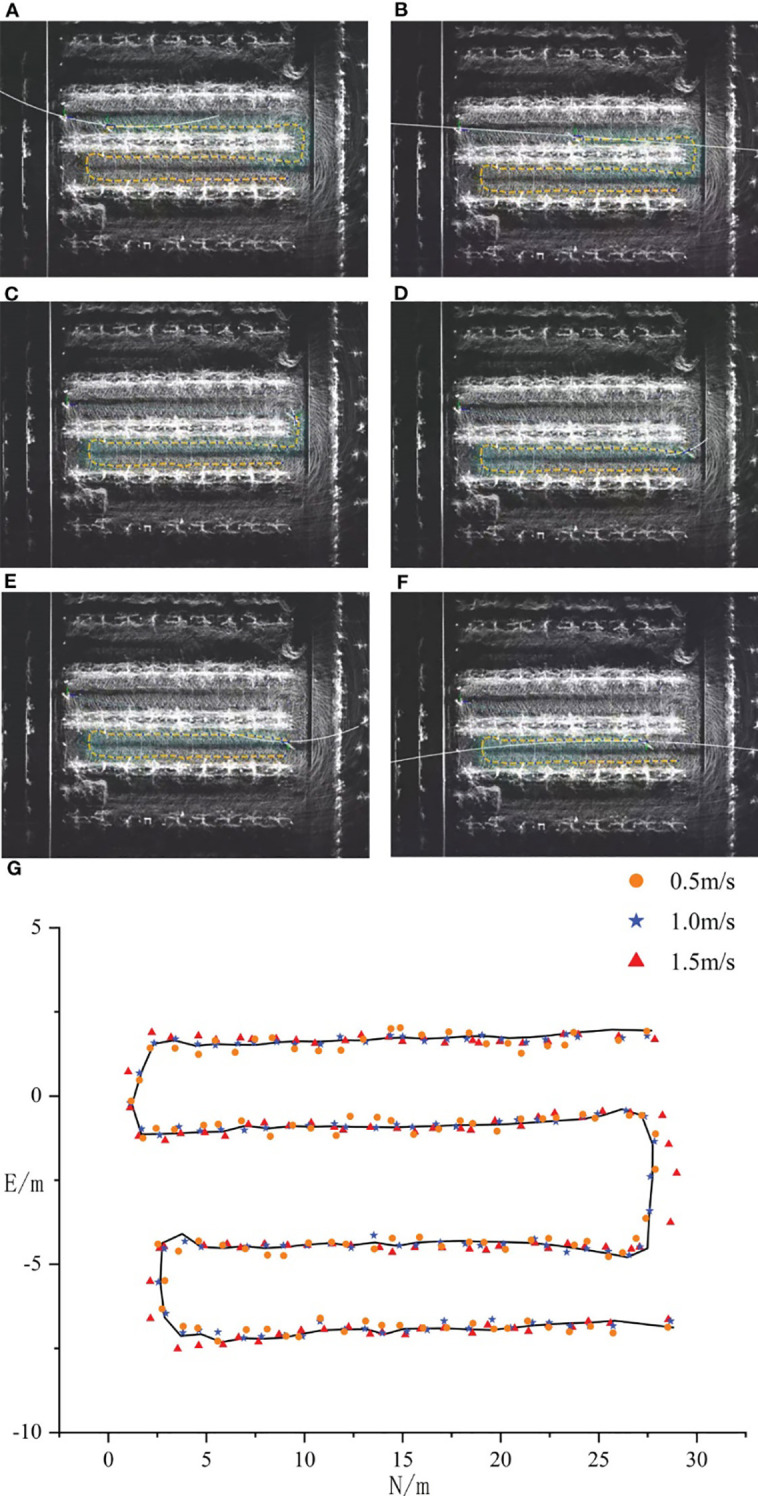
Navigation tests at different speeds (Xia et al., 2023).

Building on prior research conducted in similar orchard environments, a driving speed of 1.0 m/s was identified as the optimal speed to achieve high navigation accuracy and stability (Xia et al., 2023). This speed enables the robot to strike an ideal balance between steering responsiveness and trajectory tracking while minimizing lateral tracking errors. As shown in **Figure 12**, field tests from previous studies demonstrated that, at 1.0 m/s, the navigation system requires fewer heading adjustments and performs more smoothly on both straight and curved paths. Given the comparable experimental setup and conditions in this study, a speed of 1.0 m/s was adopted for all navigation tests.

**Figure 13:**
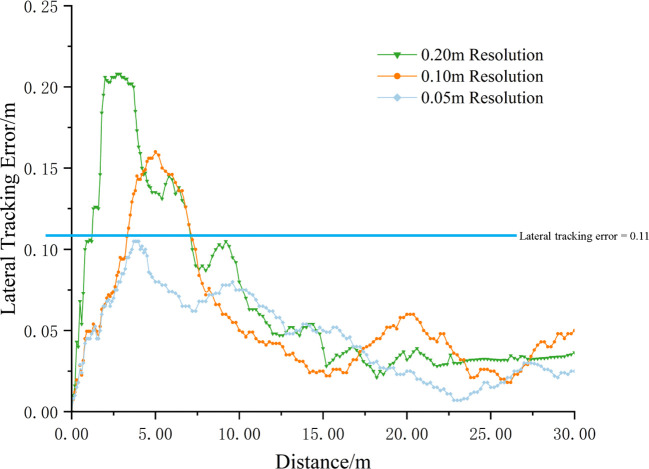
Lateral tracking errors.

The lateral tracking error of the robot, derived from real-time tracking position data provided by RTK, is illustrated in **Figure 13**. To comprehensively evaluate the tracking performance, the maximum value, average value, and standard deviation of the lateral tracking error were calculated, with the results summarized in **Table 4**.

**Table 4:** Comparison of lateral tracking errors.

Octree Resolution (m)	Maximum Value (m)	Average Value (m)	Standard Deviation (m)
0.20	0.208	0.079	0.060
0.10	0.160	0.059	0.040
0.05	0.105	0.048	0.027

When the octree node resolution was set to 0.20 m, the robot’s lateral tracking error exhibited a maximum value of 0.208 m, an average value of 0.079 m, and a standard deviation of 0.060 m. At a resolution of 0.10 m, the maximum lateral tracking error decreased to 0.160 m, the average value to 0.059 m, and the standard deviation to 0.040 m. Further increasing the resolution to 0.05 m reduced the maximum lateral tracking error to 0.105 m, the average value to 0.048 m, and the standard deviation to 0.027 m.

Overall, the robot’s tracking performance on the path improves as the resolution of the octree node increases. The reason is consistent with the previous analysis. When the resolution of the octree node increases, the generated path becomes more accurate and smoother, which facilitates the robot’s tracking operation. The drawback remains that it sacrifices planning efficiency and time to a certain extent.

## 5 Discussion

This study introduces a method leveraging the octree data structure to optimize 3D point cloud space, effectively addressing the challenges of processing large-scale 3D LiDAR maps in orchards with low computational efficiency. The experimental results demonstrate that the amount of point cloud data optimized using this method has decreased by 76.32%, effectively reducing the data scale.

To tackle the problem of low sampling efficiency of RRT in the inter-row space of orchards, this paper employs octree nodes as the unit nodes for the expansion of the random tree in the RRT algorithm and introduces an adaptive step size adjustment strategy to balance the resolution of octree nodes and the step size of the RRT algorithm, thereby generating a global path that the robot can follow. Experimental results under three resolutions (0.2 m, 0.1 m, and 0.05 m) show that this method significantly improves path planning performance compared to the traditional RRT algorithm. It reduces average path length by 9.20% and curvature by 51.16%, demonstrating enhanced path smoothness. Additionally, it increases sampling point utilization by 19.02 percentage points and improves planning efficiency with a 44.98% increase in path generation speed.

In comparison to existing methods, our approach demonstrates significant advantages in computational efficiency and adaptability. For example, Wang et al. (2022) used 3D LiDAR for high-precision obstacle detection and environmental map construction, but their approach struggled with real-time performance due to the computational burden of processing large-scale point cloud data. Similarly, Liu et al. (2023) improved path smoothness through dual-source LiDAR point clouds, focusing on combining high-frequency and low-frequency data. In contrast, our octree-based method prioritizes a balance between computational efficiency and path accuracy, making it more suitable for dynamic orchard environments. Compared to Han et al. (2021), which relied on visual navigation and required retraining for different orchard scenarios, our approach achieves robust performance across varying environmental conditions without the need for frequent algorithm updates.

If GNSS signals are subjected to dense canopy conditions, significant degradation due to multipath effects may occur, potentially leading to localization errors of up to 0.11 m. Furthermore, while the octree-based RRT* algorithm improves path planning efficiency, it could face challenges in handling dynamic obstacles, particularly during abrupt row-end turns. If irregular tree arrangements are present, the adaptive step size adjustment strategy might occasionally fail to ensure optimal path smoothness. Additionally, if the robot’s embedded hardware has computational limitations, delays in processing large-scale point cloud data could arise, potentially impacting real-time navigation performance. Future research will address these limitations by incorporating multi-sensor fusion techniques, such as integrating LiDAR data with semantic camera information, to improve obstacle detection and path planning in complex orchard environments. Additionally, we plan to optimize the computational framework and improve the adaptability of the step size adjustment strategy to overcome these challenges and ensure more robust and efficient navigation.

## 6 Conclusions

This paper proposes an autonomous navigation method for mobile robots in orchard environments based on the octree data structure and an improved RRT algorithm. By utilizing octree to optimize the 3D point cloud space, the data scale is effectively reduced. Through the introduction of octree nodes and an adaptive step size adjustment strategy, the sampling efficiency and path quality of the RRT algorithm in the inter-row space of orchards are enhanced. The experimental results demonstrate that this method can provide a technical reference for the autonomous navigation of mobile robots in orchards.

This research highlights the potential of the octree data structure to optimize 3D point cloud space and enhance path planning efficiency, contributing to the advancement of autonomous navigation technology for mobile robots in orchard environments. Furthermore, as different orchard environments exhibit unique characteristics, other researchers can adopt the ideas from this study to design experiments tailored to their specific conditions, thereby selecting optimal operational parameters. The structural characteristics of the octree not only enhance the current navigation scheme but also offer significant potential for integration with other advanced techniques such as heuristic search and pruning strategies. This scalability underscores its applicability across diverse and dynamic orchard scenarios, paving the way for broader adoption in autonomous navigation research. Future research can explore additional octree-based optimization techniques, such as heuristic search, pruning strategies, bidirectional search, and collision detection, to further improve algorithm performance and enable its application in more complex and dynamic orchard environments.

## Data Availability

The raw data supporting the conclusions of this article will be made available by the authors, without undue reservation.
